# The Influence of Local Pamidronate Application on Alveolar Dimensional Preservation after Tooth Extraction—An Animal Experimental Study

**DOI:** 10.3390/ijms21103616

**Published:** 2020-05-20

**Authors:** Frederic Kauffmann, Christian Höhne, Alexandre Thomas Assaf, Tobias Vollkommer, Jan Semmusch, Aline Reitmeier, Jamal Michel Stein, Max Heiland, Ralf Smeets, Rico Rutkowski

**Affiliations:** 1 Department of Oral and Craniomaxillofacial Surgery, Center for Dental Medicine, University Medical Center Freiburg, 79106 Freiburg, Germany; frederic.kauffmann@uniklinik-freiburg.de; 2 Department of Prosthodontics, Julius-Maximilians-University, 97070 Würzburg, Germany; hoehne_c@ukw.de; 3 Department of Oral and Maxillofacial Surgery, University Medical Center Hamburg-Eppendorf, 20251 Hamburg, Germany; a.assaf@uke.de (A.T.A.); t.vollkommer@uke.de (T.V.); j.semmusch@uke.de (J.S.); r.smeets@uke.de (R.S.); 4 Department of Laboratory Animal Science, University Medical Center Hamburg-Eppendorf, 20251 Hamburg, Germany; a.reitmeier@uke.de; 5 Department of Operative Dentistry, Periodontology and Preventive Dentistry, University Hospital Aachen (RWTH), 52074 Aachen, Germany; jstein@ukaachen.de; 6 Charité – Universitätsmedizin Berlin, Corporate Member of Freie Universität Berlin, Humboldt-Universität zu Berlin, and Berlin Institute of Health, Department of Oral and Maxillofacial Surgery, 14197 Berlin, Germany; max.heiland@charite.de; 7 Department of Oral and Maxillofacial Surgery, Division of Regenerative Orofacial Medicine, University Medical Center Hamburg-Eppendorf, 20251 Hamburg, Germany

**Keywords:** pamidronate, socket preservation, ridge preservation, bone remodeling, bone regeneration, bisphosphonates

## Abstract

The aim of this randomized, controlled animal exploratory trial was to investigate the influence of local application of aminobisphosphonate pamidronate during the socket preservation procedure. Mandibular premolars were extracted in five Göttingen minipigs. Two animals underwent socket preservation using BEGO OSS (n = 8 sockets) and three animals using BEGO OSS + Pamifos (15 mg) (n = 12 sockets). After jaw impression, cast models (baseline, eight weeks postoperative) were digitized using an inLab X5 scanner (Dentsply Sirona) and the generated STL data were superimposed and analyzed with GOM Inspect 2018 (GOM, Braunschweig). After 16 weeks, the lower jaws were prepared and examined using standard histological methods. In the test group (BEGO OSS + pamidronate), buccooral dimensional loss was significantly lower, both vestibulary (−0.80 ± 0.57 mm vs. −1.92 ± 0.63 mm; *p* = 0.00298) and lingually (−1.36 ± 0.58 mm vs. −2.56 ± 0.65 mm; *p* = 0.00104) compared with the control group (BEGO OSS). The test group showed a significant difference between vestibular and lingual dimensional loss (*p* = 0.04036). Histology showed cortical and cancellous bone in the alveolar sockets without signs of local inflammation. Adjuvant application of pamidronate during socket preservation reduces alveolar dimensional loss significantly. Further investigations with regard to dose–response relationships, volume effects, side effects, and a verification of the suitability in combination with other bone substitute materials (BSMs) are necessary.

## 1. Introduction

The osseous volume of the alveolar ridge can be lost as a result of various causes such as tooth extraction, periodontal disease, pathological bone changes, or physiological age-related atrophy processes, and may be present in the form of single- or multi-walled horizontal, vertical, or combined defects [1,2]. The dimensional changes of hard and soft tissues resulting from tooth extractions have been well studied in both animal models and humans. In this context, Tan et al. described an average horizontal bone loss of 29%–63% and a vertical bone loss of 11%–22% only six months after extraction [3]. Another research group reported on bony resorption processes with about 3.87 mm width loss and 1.67 to 2.03 mm height loss, especially in the first three months after tooth extraction [4]. It could be shown that the strongest changes of the alveolar process occur within three to six months after tooth extraction and manifest primarily at the buccal alveolar wall [5]. As a possible cause for the buccal pronounced resorption processes, the bundle bone predominantly occurring in the crestal area of the alveolar ridge is discussed [6,7]. This bone, which is nurtured primarily via the periodontal ligaments, inevitably loses its nutrition even after atraumatic extraction, which promotes resorption mediated by osteoclasts. A further predictor for the resorption processes is the thickness of the buccal alveolar wall. In this context, Chappuis et al. used cone-beam computed tomography to show that a vestibular bone thickness of <1 mm results in significantly higher vertical bone loss than a vestibular bone thickness of >1 mm [8]. The atrophy and remodeling processes result not only in a loss of volume, but also in a displacement of the alveolar ridge towards the lingual or palatinal side, which results in both aesthetic and, with regard to masticatory rehabilitation, functional deficits. In this context, socket preservation was born as a procedure to prevent or, more properly, limit the alteration of the post-extraction bone crest as a function of an optimal implant–prosthetic rehabilitation. Augmentative procedures have been established for several decades in dentoalveolar surgery and represent a crucial component of individual functional oral and masticatory rehabilitation in state-of-the-art dentistry [9,10,11]. In addition to autologous bone, which remains as the gold standard owing to its osteoinductive characteristics, various bone substitute materials (BSMs) are used to stabilize the alveolar socket after tooth extraction, whereby basically human donor materials (allogeneic), animal (xenogeneic), and synthetic substitutes (alloplastic) are differentiated [12]. With an overall manifold spectrum of indications, all BSMs have in common that they should serve as a basic framework with an individually pronounced pore structure as a guide rail for cellular and subcellular structures responsible for the processes of bone formation in vertical and horizontal dimensions and re-vascularization. However, allogeneic, xenogeneic, and alloplastic bone graft substitutes show significant differences to the biomechanical and immunological properties of autologous bone. The simultaneous application of membranes and bone replacement materials in the sense of guided-bone regeneration (GBR) for the prevention of resorption showed additional positive effects for the preservation of the vertical and horizontal structures of the alveolar process [13,14,15]. The use of biological agents such as rhBMP2 (recombinant human bone morphogenic protein 2), bFGF (basic fibroblast growth factor), rhPDGF (recombinant human platelet derived growth factor), and TGF-b (transforming growth factor beta) had also been shown to promote osteogenic induction in socket preservation in recent studies [16,17]. In addition, the additive use of autologous platelet concentrates (APCs) as a source of various growth factors is also becoming increasingly popular in the context of socket preservation (SP) [18,19,20]. Although some beneficial effects in terms of dimensional changes were observed, none of the socket preservation techniques could entirely prevent bone resorption [21,22]. In addition, statistically significant differences in new bone formation and dimensional changes both between the different bone graft substitutes and compared with spontaneous healing are rare [23]. Modulating the bone response and inhibiting osteoclast activity, bisphosphonates (BPs) are commonly used to treat osteoporosis, Paget’s disease, bone metastasis, multiple myeloma, and primary hyperparathyroidism [24,25,26,27,28,29]. Although BPs’ systemic use may cause side effects as bisphosphonate-related osteonecrosis of the jaw (BRONJ) or esophagitis, topical application appears to have a positive effect on the health of the periodontium and bone formation [30,31]. In orthopedics and traumatology, local administration of BPs has been shown to promote bone formation and reduce bone resorption [32,33]. There are also reports in implant dentistry showing that BPs contribute to peri-implant bone formation and improve fixation of osseointegrated implants [34,35,36]. In an animal study, Möller et al. showed a reduced surface resorption of autologous onlay grafts using alendronate in aqueous solution (1 mg/mL) in three different ways, on a collagen membrane, soaked in bovine bone mineral granules, or applied to the bone graft directly [37]. Local treatment may be advantageous compared with systemic use as the amount of BP at the site of surgery is (at least initially) more controllable [38]. The aim of this animal study was to investigate the influence of the local application of pamidronate (aminohydroxypropylidine bisphosphonate, APD) during socket preservation using xenogenic bone substitution material on the resorptive remodeling processes and the dimensional preservation of alveolar structures after premolar extraction in Göttingen minipigs, hypothesizing that APD reduces alveolar dimensional loss.

## 2. Results

The surgical procedure could be performed in all animals without complications. In the follow-up period, all animals showed stage-appropriate wound healing without clinical evidence of a local inflammatory reaction or wound dehiscence, especially 8 and 16 weeks after surgery. At the beginning of the study, the weight of the pigs was between 100 and 107 kg (normally distributed). A substantial change in weight after surgery was not observed during standardized animal housing. To exclude possible differences in jaw size despite equal age and normally distributed body weight, the width of the alveolar ridge of the jaws was measured by means of the scans, whereby a normal distribution (*p* = 0.878) was detected using the Kolmogorov–Smirnov test.

### 2.1. Cast Models

**Figure 1:**
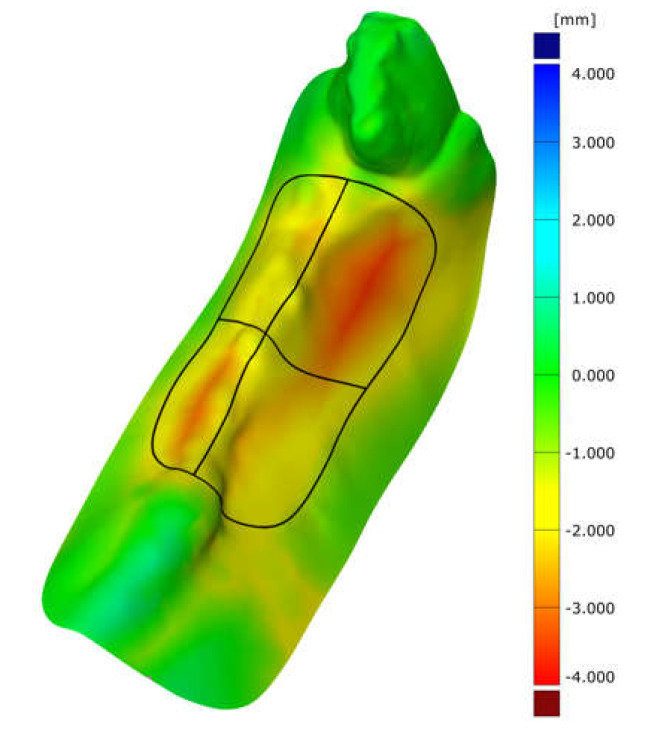
Superimposition of two scans (preoperatively and eight weeks after tooth extraction) shows the dimensional changes over the marked areas (P2/P3 buccal and oral area) in a false-color image. Negative values represent a decrease in area and consecutive reduction in volume. An increase in the volume of the alveolar area is not visible. The black line marks the area of interest and its separation into the buccal and lingual part. The green area surrounding the alveoli illustrates the high congruence in the non-operated areas and proves the exact superposition of the models and the scans, respectively.

After all cast models were scanned, it was not possible to superimpose the data directly after surgery (post OP) and the 16-week endpoint data (after sacrifice) owing to insufficient impression quality and resulting incorrect scans. The scans of the baseline casts (before surgery) and those of the impressions taken eight weeks after surgery could be analysed and superimposed (Figure 1).

### 2.2. Superimposition Analysis

**Figure 2:**
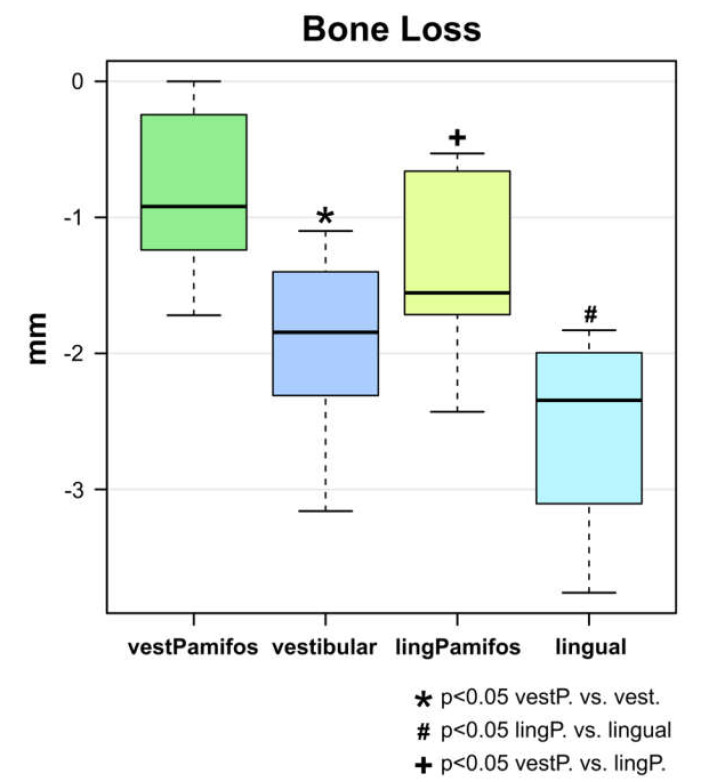
Boxplot analysis of the surface dimensional changes eight weeks after tooth extraction and socket preservation. The bucco-oral dimensional loss was significantly lower in the test group (BEGO OSS + pamidronate) compared with the control group (BEGO OSS) both buccal (−0.80 ± 0.57 mm vs. −1.92 ± 0.63 mm; *p* = 0.00298) and oral (−1.36 ± 0.58 mm vs. −2.56 ± 0.6 mm; *p* = 0.00104). The test group also showed a significantly increased loss of dimension in the buccal aspect compared with the oral aspect of the alveolar process (*p* = 0.04036).

GOM Inspect 2018 (GOM, Braunschweig, Germany) was used to superimpose the generated STL data (baseline and eight weeks post-surgery). In the test group (BEGO OSS + pamidronate), bucco-oral dimensional changes were significantly lower in both the buccal as well as the oral aspect than in the control group (BEGO OSS) (Figure 2). The dimensional changes in the test group in the buccal aspect were −0.80 ± 0.57 mm and −1.92 ± 0.63 mm in the control group, respectively (*p* = 0.00298) (Figure 2). In the oral aspect in the test group, the dimensional changes were −1.36 ± 0.58 mm and −2.56 ± 0.65 mm for the control group, respectively (*p* = 0.00104). The overall difference between the two groups was 58% in the buccal and 47% in the lingual aspect, respectively. Additionally, the test group also showed a significant difference between vestibular and lingual dimensional loss (*p* = 0.04036) (Figure 2).

### 2.3. Histology

**Figure 3:**
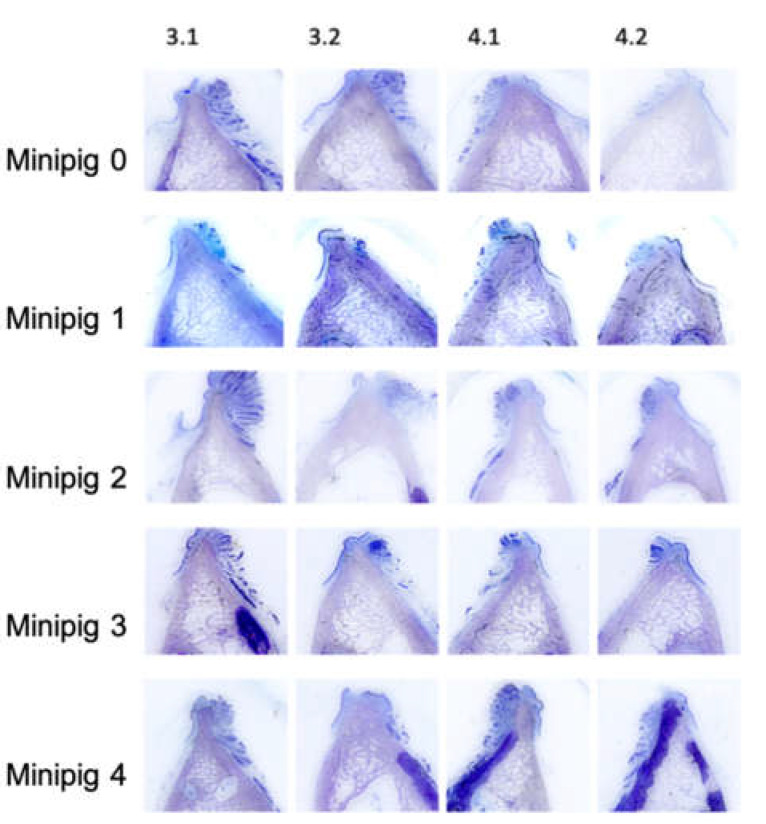
Overview of the thin-section histologies of all former extraction sockets, two premolars in the mandible on both sides (staining: toluidine blue; magnification: 2×).

**Figure 4:**
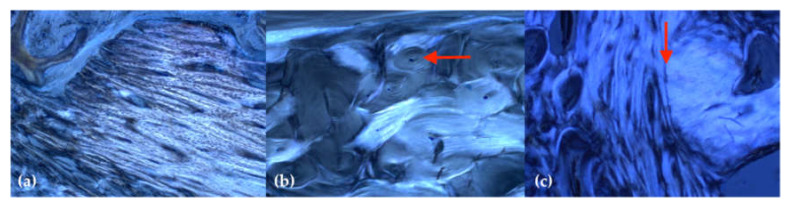
Representative imaging of the bone structure in polarized light (staining: toluidine blue; magnification: 50×): (**a**) primarily parallel aligned, newly formed bone structure in the former tooth socket; (**b**) the remaining cortical bone shows a typical osteon structure (red arrow); (**c**) border (red arrow) between the original bone tissue (right) and new bone tissue resulting from remodelling (left).

All extraction sockets showed complete healing after 16 weeks without signs of local inflammation or disturbed wound healing. The thin-section histologies showed that all extraction sockets—with and without the application of Pamifos—are almost completely built up with bone after 16 weeks (Figure 3). Cortical and spongious bone structure as well as active bone accumulation by osteoblasts were found in the extraction sockets. This bone structure differed significantly from the surrounding cortical bone of the lower jaw. In the former sockets, primarily, lamellar structures were found, whereas the surrounding area was dominated by osteons (Figure 4).

**Figure 5:**
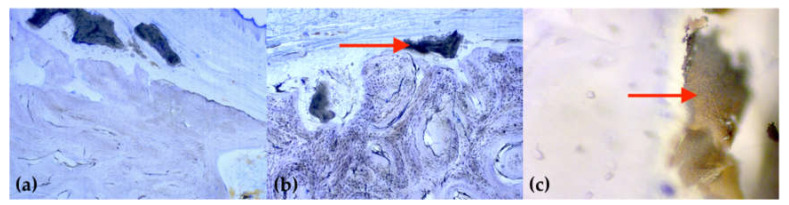
Exemplary illustration of attached or embedded bone substitute material: (**a**,**b**) isolated BEGO OSS particles (marked by arrow) are still found sporadically on the surface of all animals (staining: toluidine blue; magnification: 25x); (**c**) in cancellous bone, foreign material can only be observed in a very limited quantity (staining: toluidine blue; magnification: 400x).

Almost no BSM granules encapsulated with bone tissue could be detected in the extraction sockets. Only a few remnants of the bone substitution material were still visible in the spongiosa, whereby a reliable allocation was difficult owing to the small quantity. In almost all samples, parts of the BEGO OSS granules were still visible as an external layer on the bone. Several BEGO OSS particles showed a bone apposition, but predominantly, a connective tissue layer between the remaining BEGO OSS particles and the bone surface (Figure 5). There were no significant differences between the test and control groups in this respect.

The cortical thickness in the area of the extraction sockets varied intra- and interindividually. The mucosal coverage in the area of the augmented extraction sockets also appeared regular, with the exception of local discrete thickening in the area of BEGO OSS residues overlying the bone. Neither the cortical nor the mucosal thickness showed significant differences between the test and control group.

## 3. Discussion

The study aimed to show effects of the local application of pamidronate in combination with particulated xenogenic bone substitute material on the healing of fresh extraction sockets in an animal model (Göttingen minipig). In this context, the use of pamidronate was able to reduce post-extractional bone loss in the lingual as well as buccal aspect, resulting in a significantly reduced alveolar dimensional loss compared with the control group. Histology showed extensive bony remodeling after 16 weeks without signs of inflammation or disturbed wound healing in both groups.

The post-extractional remodeling processes are well studied and histological and volumetric results show significant bone loss, especially in the first three months after extraction. Bone loss in the buccal and lingual aspect is not equally distributed—bone loss in the buccal aspect is 2/3 versus 1/3 in the lingual aspect [39]. This may cause significant aesthetic problems and require bone and soft tissue augmentation procedures before or during implant placement. As patient morbidity is increasing with every surgery and aesthetic outcome in the anterior area is more and more compromised, a central clinical focus is on preventing bone loss after tooth extraction. Recent systematic reviews of alveolar socket preservation have concluded that various techniques, including bone graft/substitute implantation, can reduce bone loss after tooth extraction compared with spontaneous alveolar healing, but that some loss of alveolar ridge height and especially width seems unavoidable [23,40,41]. Under physiological conditions, osteoclasts are mainly responsible for the vertical and horizontal loss of alveolar bone in the area of the extraction socket [42]. The loss of bundle bone caused by disruption of the vascular blood supply from the periodontal ligament and accompanying catabolic changes cause significant osteoclastic activity [43].

Consequently, various research groups focused on the suppression of osteoclast activity by bisphosphonates. Owing to their mechanism of action, which induces an inhibition of osteoclasts, but is simultaneously associated with continued osteoblast function, bisphosphonates (e.g., alendronate, pamidronate, and zoledronate) intervene pharmacologically in the bone remodeling process, even after tooth extraction [44]. A systemic application of these drugs, usually either orally or intravenously, would probably not be easily accepted by patients, but also does not seem to be appropriate owing to the well-known systemic and local side effects [45,46]. However, bisphosphonates can also be applied topically and inhibit local bone resorption, which has been shown in various in vivo studies.

Yaffe et al. [47] showed that a local application of bisphosphonate on the outer surface of the alveolar bone can reduce the resorptive processes even on the periodontal (inner) side following mucoperiosteal flap surgery in rats. Most research groups investigated a topical application of bisphosphonates via different carrier materials, comparable to the present study. Möller et al. [37] investigated the influence of bisphosphonate on the surface resorption of bone grafts by topical application using different carrier materials. The authors showed that statistically significantly lower loss in graft height was seen on the test side for Bio-Gide^®^ + alendronate (0.65 %) versus Bio-Gide^®^ (1.52 %), *p* = 0.002; Bio-Oss^®^ + alendronate (1.16 %) versus Bio-Oss^®^ (4.20 %), *p* = 0.001; and bone graft + alendronate (1.25 %) versus bone graft alone (6.01 %), *p* = 0.006. In addition, the test groups showed a significantly improved osseointegration of the titanium screws fixing the bone graft. However, five of the alendronate-treated transplanted bone grafts showed no osseointegration and necrosis of the overlying periosteum and were subsequently excluded from the evaluation. Even in the remaining BP treated bone grafts, there were occasional signs of osteochemonecrosis after a 12-week observation period. In addition, an inhibitory effect on bone remodeling was observed by a statistically significantly lower number of resorption lacunae [37]. Astrand and Aspenberg described a similar picture of BP-associated necrosis after local application of bisphosphonates in rats [48]. In contrast, animals in our study showed no signs of local wound healing disorder or drug-associated pathologies, neither macroscopically nor microscopically.

Fischer et al. [49] reported on the local application of pamidronate for the healing of extraction sockets in dogs in a proof-of-principle study in vivo and described a delayed healing in sites that received pamidronate and a BP-inhibited resorption of the bone graft substitute introduced into the alveoli. In addition, however, this study showed a clinically potentially relevant reduction in horizontal bone loss (3 mm below the cemento-enamel junction) in the test group (Osteobiol Gen-Os + pamidronate (90 mg/mL)) compared with the control group (Osteobiol Gen-Os + sterile saline). However, a specific histomorphometric analysis regarding the percentage of residual graft, new bone, and connective tissue was not performed owing to the small number of sections. While the findings regarding the affectable post-extraction dimensional changes are thus similar to those of the present study, there are clear differences in the general healing and resorption of the bone substitute material, despite histological analysis being carried out at the same time (16 weeks after surgery) and a comparable type of bone graft material being used. In comparison, the Göttingen minipig used in this study appears to have improved wound healing.

Comparing the effects of porcine-derived bone substitution material with or without pamidronate application, it was reported that the addition of pamidronate enhances new bone formation four and eight weeks after tooth extraction in Fox hound dogs [50]. Cha et al. [51] investigated the anti-resorptive effect of pamidronate on extraction socket wall in six dogs and concluded that the cellular membrane (loaded with pamidronat) used significantly inhibited the buccal bone plate resorption at 12 weeks, resulting in diminished vertical and horizontal changes of the residual alveolar ridge. Signs of disturbed wound healing were observed in one animal from each of the test and control group, thus no causal relationship with local bisphosphonate application could be demonstrated. In a comparable approach, Saulacic et al. [52] also investigated the effects of a local application of bisphosphonate (alendronate, ALN) on bone remodeling after tooth extraction in the dog model. The authors concluded an ALN treatment is beneficial for the preservation of the lingual bone and formation of new bone within the extraction sockets. There was no significant relation between the various ALN dosages and local effect power. In contrast to the majority of comparable studies focusing on the BP effects on post-extraction socket preservation, Saulacic et al. [52] also described increased new bone formation (as well as increased porcine xenograft replacement). In one of the few works on this topic in vivo on humans, De Sarkar et al. [53] evaluated the local effects of collagen sponge soaked in alendronate in a clinical study (20 patients) and showed a significant prevention of ridge resorption four months after teeth extraction (test group: 44.38%; control group: 22.8%). With respect to the results presented here, the combination of a reduced alveolar dimensional loss and an uneventful bone apposition with at least subtotal degradation of the xenogenic bone substitution material seems to be conflicting. Although the reduced osseous dimensional loss in comparison with the control group is consistent with the previously known pharmacological mechanisms of action of bisphosphonates, a reduced resorption of the particulate bone substitution material would have been expected in this context as well. In addition to an increased bony remodelling capacity in pigs compared with humans, it must be taken into account that a descriptive histological evaluation was primarily carried out in a limited number of cases, which ultimately does not allow any evidence-based statements to be made regarding the final degree of degradation of the bone substitution material.

Summarizing the data published so far, especially in vivo animal studies, a heterogeneous picture emerges with regard to the complex subject of a possible beneficial influence on the preservation of alveolar ridge by local application of bisphosphonates after tooth extraction. The present study demonstrated beneficial effects of local application of bovine bone substitution material treated with pamidronate on post-extraction bone resorption. In this context, the innovative scanning and superimposing technology enabled, for the first time, to acquire data on the planar dimensional changes exceeding the actual alveolar borders, which further enrich current discussions. However, the results are limited (exploratory study) owing to the small number of animals, n = 5 (test: n = 3 (12 alveoli) and control: n = 2 (8 alveoli)) and lack of cone-beam computed tomography (CBCT) data. Furthermore, the almost complete osseous recovery in both groups and the absence of significant histological differences cannot be easily transferred to other time points. While various research groups were able to show a reduction of bone resorption after tooth extraction by local application of bisphosphonate, there are much more controversial data regarding aspects of local bone formation and potential drug-associated side effects/complications.

In the context of translational research, however, the well-known side effects, which clinically may include necrotic, exposed bone, pathological fractures, and recurrent soft tissue infections with significant impairment of quality of life, must be considered. In view of this highly complex risk potential related to the systematic use of antiresorptive drugs, further investigations in animal models seem to be without alternative prior to systematic studies in humans. The focus should be on the dose–response relationship, the most suitable time of application as well as type of application (direct vs. via carrier mediated such as bone substitution material or membranes) and the differentiated volumetric analysis of horizontal and vertical remodeling processes, as well as with regard to different defect forms. Furthermore, in order to achieve the best possible transferability of the data and to perform corresponding studies in humans, it is essential to supplement long-term data concerning potential side effects.

## 4. Materials and Methods

The study was designed as a randomized, controlled experimental study. Owing to the sample limitations, the study design is intended to be exploratory. It conforms to the Guide for the Care and Use of Laboratory Animals, eighth edition, updated by the U.S. National Research Council Committee in 2011 and was performed in accordance with the European Directive 2010/63 EU. The research protocol was approved by the local animal research committee (Project Registration No. 119/12; Date of approval: December 2012).

### 4.1. Animals—General Information

All experiments were performed on five healthy, full-grown Göttingen minipigs aged 48–50 months, bred for laboratory animal use (G. Schlesier, Dresden, Germany). None of the minipigs had been previously used in other studies and none were excluded from the present study. The minipigs were kept in one group. The diet consisted of straw, hay, water ad libitum, and a limited amount of special minipig diet (Ssniff Spezialdiäten GmbH, Soest, Germany) twice per day. The minipigs were adequately supplied with fresh bedding material on a daily basis. There was a 12 h light and dark cycle, a temperature of 18 ± 2 °C, and relative humidity between 50% and 60% [54]. The housing was open with no special hygienic requirements. There was a quarterly hygienic monitoring of endo- and ectoparasites according to the FELASA recommendations. Housing conditions were not changed during the experiment. The minipigs were frequently monitored on a daily basis during cleaning and feeding times. Post-surgery, they were checked at least once per day by the responsible veterinarian.

### 4.2. Animals—Anesthesia and Surgery

Animals were fasted 16 h before surgery. Anesthesia was initiated with intramuscular injections of 0.2 mg/kg midazolam (Roche Pharma AG, Grenzach-Wyhlen, Germany), 10 mg/kg Ketamine (WDT, Garbsen, Germany), and 4 mg/kg stresnil (Elanco Deutschland GmbH, Bad Homburg, Germany). A venous catheter was placed into the auricular vein. The animals were intubated after administering 2–4 mg/kg propofol 1 %v (B. Braun, Melsungen, Germany) intravenously. Anesthesia was maintained with 1.5% isoflurane (Baxter Deutschland GmbH, Unterschleißheim, Germany). The animals were ventilated with a mixture of air and 35% oxygen (Zeus, Drägerwerk, Lübeck, Germany) and placed on a warming mattress (Moeck & Moeck GmbH, Germany) to prevent hypothermia and maintain the body temperature between 37.5 °C and 38 °C. During procedures, ECG and oxygen saturation monitoring as well as noninvasive blood pressure measurements (Infinity Delta monitor, Drägerwerk, Lübeck, Germany) were performed. At the beginning of the procedure, preoperative analgesics (4 mg/kg carprofen (Zoetis Inc., Florham Park, NJ) and 50 mg/kg metamizole (WDT, Garbsen, Germany)) were administered intravenously. Fentanyl (Janssen-Cilag, Neuss, Germany) was administered via constantly perfusion (Perfusor^®^ Space B.Braun, Germany) between 5 and 20 µg/kg/h during surgery. An antibiotic prophylaxis of 10 mg/kg clindamycin (WDT, Garbsen, Germany) was administered intravenously pre-operative.

To ensure a sufficient analgesia, a vestibular and oral local infiltration of 5 mL of Ultracain^®^ 4% with adrenaline 1: 100,000 (Sanofi-Aventis GmbH, Frankfurt, Germany) was performed. Afterwards, mandibular premolars were extracted in five Göttingen minipigs. The extraction of all teeth was performed using a piezo with a dental unit and a specialized extraction kit (Piezosurgery; Mectron Medical Technology, Carasco, Italy). All teeth were initially loosened by the piezo and afterwards extracted using conventional tooth extraction instruments, such as Bein’s tooth elevator and tooth extraction pliers. In all cases, extractions were gently performed to protect the alveolar sockets. After extraction, two animals underwent socket preservation using BEGO OSS (n = 8 sockets) and three animals using BEGO OSS + Pamifos (15 mg) (n = 12 sockets). The xenogen BSM was soaked with 5 mL Pamifos^®^ (3 mg/mL concentrate) for 10 min and the mixture was subsequently applied into the extraction socket. In all animals, a buccal mucoperiosteal flap was used to close the alveolar extraction sockets. Wound closure was performed by simple interrupted sutures using resorbable 3-0 Vicryl RAPIDE UNDYED 1x36" V-34 (Ethicon, Johnson & Johnson, USA).

All animals recovered quickly within a maximum of 120 min after the surgery and showed no signs of pain or impairment (restrictions of food intake, movement, and curiosity) on the following day. The post-operative treatment consists of 4 mg/kg Carprofen (Zoetis Inc., Florham Park, NJ) once per day and 10 mg/kg clindamycin (WDT, Garbsen, Germany) twice per day orally for ten days. After 16 weeks, all five minipigs were sacrificed by intravenous applications of T61 (200 mg embutramide, 50 mg mebezonium, and 5 mg tetracaine per ml; 6 mg/50 kg) after deep sedation and the mandibles were surgically removed.

### 4.3. Dental Models, Scanning Procedure, and Digital Analysis

The cast models made based on the intraoral impressions at the defined time points (baseline, immediately after surgery, 8 and 16 weeks after surgery) were optically scanned using InEos X5 scanners (Dentsply Sirona, York, Pennsylvania, USA), and corresponding STL files were created. The STL files of the cast models were automatically aligned by computational best-fit alignment algorithms of GOM Inspect 2018 (GOM, Braunschweig, Germany), superimposing the non-moving tooth structures visible in the optical scans and saved. GOM Inspect is used by the industry for product development, quality control, and production with a focus on alignment and deviation measurement. The software is certified by NIST (National Institute of Standards and Technology, Gaithersburg, Maryland, United States) and PTB (Physikalisch-Technische Bundesanstalt, Braunschweig und Berlin). GOM Inspect has been placed in Category 1 with the smallest measurement deviations.

**Figure 6:**
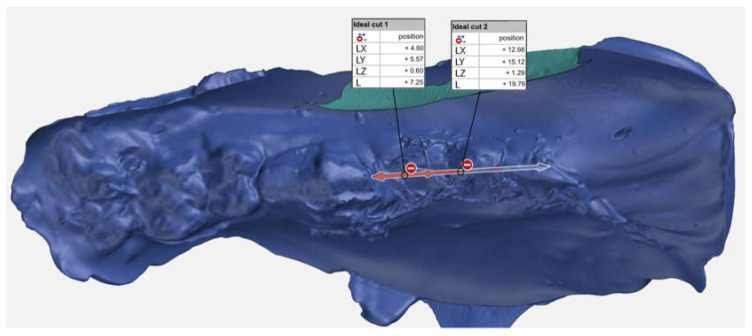
Scan of the immediate postoperative impression with metric analysis of the exact position of the centre of the extraction sockets (shown by the arrows) for later placement of the histological sections.

Regions of interest (P2/P3 bone crest 8 mm towards the buccal as well as to the oral aspect) were marked, and dimension changes were calculated. To determine an exact and consistent area of interest in the buccal and oral aspect of the extraction site, the buccal area was defined as follows: the exact crestal midline beginning distal of the canine and 8 mm perpendicular towards the vestibulum. The oral area was defined as the same crestal midline, but 8 mm to the oral (Figure 6). To make sure samples for histology were cut and analyzed exactly in the center of each socket, measurements following STL data were created to ensure the correct position (Figure 6).

GOM inspect was used to measure the dimensional changes in the buccal and oral ridge area comparing the dimensions before tooth extraction (baseline) and eight weeks after socket preservation. The analysis was not a single point selection. All measurements from the STL file were considered. This resulted in more precise information about the mean surface deviation. As the selected area is not exactly the same consistent 3D position and if only single point probes were done, there would have been a greater deviation in the results. The mean volume change per area was calculated as a distance in buccal and oral direction to allow a direct comparison of dimensional changes between the sites.

### 4.4. Histological Analysis

The jaws taken 16 weeks after tooth extraction and augmentation were fixed in 4% formaldehyde and a contact X-ray and photo documentation was performed. The jaws were then separated in the area of the extracted teeth using a diamond band saw (EXAKT, Norderstedt, Germany). The removed jaw segments with a thickness of 4 mm were processed into decalcified thin section preparations [55].

For this purpose, the bone segments were first dehydrated and degreased by an ascending alcohol series in a carousel-type tissue processor (Citadel 2000, Shandon, Frankfurt am Main, Germany). Subsequently, infiltration into polymer (Technovit 7200 VCL (Heraeus-Kulzer GmbH & Co. KG, Wehrheim, Germany) and then light polymerisation were performed. Using an automatic grinding system (EXAKT micro-grinding system 400 CS, EXAKT Apparatebau, Norderstedt, Germany), thin section preparations with a thickness of 45 µm were prepared from the embedded slides and stained toluidine blue for histological analysis.

### 4.5. Statistical Analysis

Data were summarized in terms of means and standard deviations. The dimensional changes before and after tooth extraction using Pamidronate were compared for the two different groups at time points (before surgery vs. 8 weeks). Statistics analysis was performed with a 95% confidence interval (5% risk) using Mann–Whitney U test with a statistical software (SPSS version 25, IBM, Armonk, NY, USA). The results of tests with *p*-values ≤ 0.05 were considered as statistically significant.

## 5. Conclusions

In conclusion, the hypothesis of this study was accepted that a bisphosphonate-treated bovine bone substitute material significantly reduced post-extraction alveolar dimensional loss compared with a non-bisphosphonate-treated control group. The used technique (superimposing, no single point analysis) helps to reduce the bias (even if blinded examiners) risk. This can and will lead to better comparable results when compared with single point analysis. The data emphasize the importance of preclinical testing and show that further studies are needed to improve the technique of local BP application and to optimize essential aspects such as the dose–effect relationship, indication specificity, and carrier options, among others, and to complement clinical long-term data.

## Abbreviations

BSM	Bone substitute material
GBR	Guided bone regeneration
rhBMP2	Recombinant human bone morphogenic protein 2
bFGF	Basic fibroblast growth factor
rhPDGF	Recombinant human platelet derived growth factor
TGF-b	Transforming growth factor beta
APC	Autologous platelet concentrates
SP	Socket preservation
BP	Bisphosphonate
BRONJ	Bisphosphonate-related osteonecrosis of the jaw
APD	Pamidronate
ALN	Alendronate
CBCT	Cone-beam computed tomography
